# Heart Rate Variability as a Tool for Seizure Prediction: A Scoping Review

**DOI:** 10.3390/jcm13030747

**Published:** 2024-01-27

**Authors:** Federico Mason, Anna Scarabello, Lisa Taruffi, Elena Pasini, Giovanna Calandra-Buonaura, Luca Vignatelli, Francesca Bisulli

**Affiliations:** 1 Department of Biomedical and Neuromotor Sciences, University of Bologna, 40126 Bologna, Italy; federico.mason@unibo.it (F.M.); anna.scarabello@studio.unibo.it (A.S.); lisa.taruffi@studio.unibo.it (L.T.); giovanna.calandra@unibo.it (G.C.-B.); francesca.bisulli@unibo.it (F.B.); 2 IRCCS Institute of Neurological Sciences of Bologna, Full Member of the European Reference Network EpiCARE, 40139 Bologna, Italy; elena.pasini@isnb.it

**Keywords:** seizure detection, seizure prediction, heart rate variability, epilepsy

## Abstract

The most critical burden for People with Epilepsy (PwE) is represented by seizures, the unpredictability of which severely impacts quality of life. The design of real-time warning systems that can detect or even predict ictal events would enhance seizure management, leading to high benefits for PwE and their caregivers. In the past, various research works highlighted that seizure onset is anticipated by significant changes in autonomic cardiac control, which can be assessed through heart rate variability (HRV). This manuscript conducted a scoping review of the literature analyzing HRV-based methods for detecting or predicting ictal events. An initial search on the PubMed database returned 402 papers, 72 of which met the inclusion criteria and were included in the review. These results suggest that seizure detection is more accurate in neonatal and pediatric patients due to more significant autonomic modifications during the ictal transitions. In addition, conventional metrics are often incapable of capturing cardiac autonomic variations and should be replaced with more advanced methodologies, considering non-linear HRV features and machine learning tools for processing them. Finally, studies investigating wearable systems for heart monitoring denoted how HRV constitutes an efficient biomarker for seizure detection in patients presenting significant alterations in autonomic cardiac control during ictal events.

## 1. Introduction

Epileptic seizures represent the main burden for People with Epilepsy (PwE), who, because of ictal events, may suffer physical injuries, loss of consciousness, and, in the most dangerous cases, status epilepticus and Sudden Unexpected Death in Epilepsy (SUDEP) [1]. The unpredictability of seizures leads PwE to experience anxiety and depression, significantly affecting their quality of life (QoL) [2]. In this context, the implementation of real-time warning systems that can detect or predict seizures would enhance the management of these episodes, leading to high benefits to both PwE and their caregivers [3].

In the past decades, it has been recognized that PwE present a complex interaction between brain and heart. Some studies have suggested alterations in autonomic activity following focal seizures spreading into cortical structures such as the amygdala, insula, and cingulate gyrus, with possible involvement of the thalamus and hypothalamus [4]. In Temporal Lobe Epilepsy (TLE), ictal events often correlate with tachycardia, which may precede seizure onset by seconds to minutes [5]. At the same time, in some cases, ictal bradycardia can also be observed [6]. In general, it remains challenging to assess the role of cardiovascular dysregulation during the peri-ictal phase and its interaction with sympathetic/parasympathetic balance.

Heart rate variability (HRV), defined as the variability of the intervals among consecutive heartbeats, is one of the most popular tools for estimating the balance between sympathetic and parasympathetic tones [7,8]. Fluctuation in the inter-heartbeat intervals is considered a protective mechanism to respond to sudden cardiovascular demands, and a decreased HRV can be linked to an elevated risk of sudden cardiac death [9]. Differently from other cardiovascular parameters, HRV can be recorded using a single-lead electrocardiogram (ECG), and, consequently, can be monitored through noninvasive wearable devices, like wrist-worn smartwatches or chest-strap sensors [10,11].

In the literature, multiple studies have reported changes in cardiac autonomic control before seizure onset, suggesting that the HRV could be a predictive biomarker of ictal events [5,12,13,14,15,16]. Such evidence and the relative easiness of HRV recording have encouraged the development of HRV-based seizure detectors, with the dual aim of improving QoL and minimizing the risk of sudden death for PwE [17]. Despite presenting a lower accuracy than more complex systems that integrate features of different natures, HRV-based systems are associated with high usability and acceptability levels, enabling continuous health monitoring even outside a clinical context [18]. To date, no detection systems have provided accurate results over broad populations and there is no agreement on which HRV-based features could better assess ictal events.

This manuscript consists of a scoping review of the literature analyzing HRV modifications during the peri-ictal phase and aims to provide new insights on how to develop efficient systems for predicting seizures. Despite other reviews having been written in this field [5,19,20,21,22,23,24], to the authors’ knowledge, these previous works did not focus on analyzing how HRV changes during the ictal transitions but generally compared cardiac modifications between different populations or associated HRV modification with seizure severity. The most similar work in the literature is [25], which presents significant methodological limits since it does not follow systematic criteria for selecting the papers and comparing their content.

## 2. Materials and Methods

### 2.1. Search Strategy

Our review followed the Preferred Reporting Items for Systematic Reviews and Meta-Analyses (PRISMA) guidelines [26]. The PubMed database was systematically searched for original studies investigating HRV changes during ictal periods, considering all the works published from January 1980 to 30 June 2023. In carrying out the initial research, a combination of synonyms of “Epilepsy” and “Heart Rate Variability” were considered as search terms (see Table 1).

**Figure 1:**
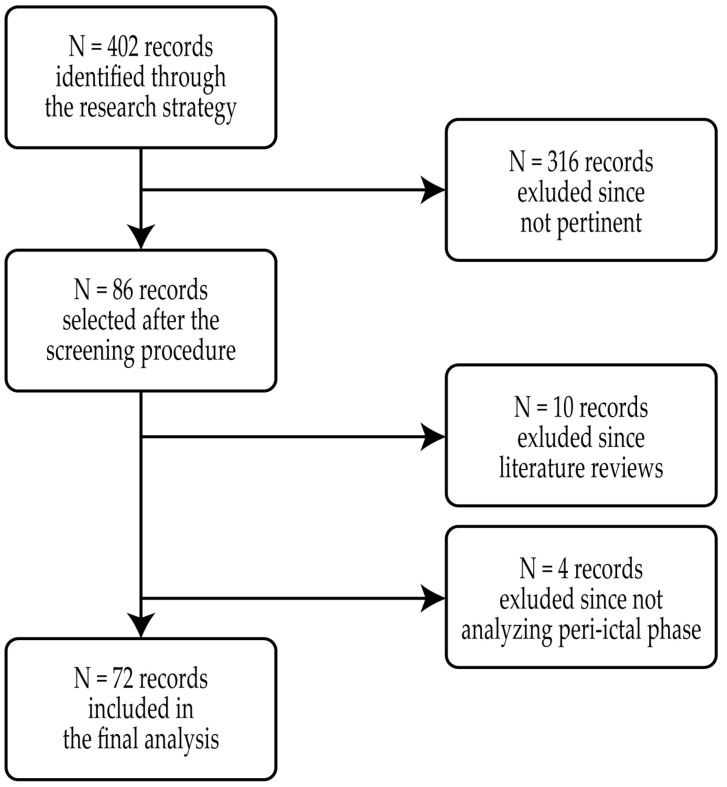
Flow chart of the selection procedure.

When selecting the papers to be included in the review, the following criteria were considered: being written in English; considering PwE as the studied population; analyzing HRV changes during the peri-ictal phase; and developing seizure detection tools taking HRV measures as an input. A double-screening approach was followed where the abstract of each record was independently analyzed by two authors (F.M. and A.S.). If there was no concordance on discarding or including the record in the final selection, a third author (L.T.) was asked to assess if the abstract complied with the review criteria. After the screening, the full text of each record selected was reviewed by three authors (F.M., A.S., and L.T.) to assess its eligibility for the final analysis. A flow chart of the full selection procedure is given in Figure 1.

The following information was collected from each study: the heartbeat recording, the types of HRV metrics analyzed, the algorithms implemented for classifying ictal periods, the characteristics of the studied population, the clinical outcomes derived from the HRV analysis (comparing different demographic groups or seizure types), and the performance of the systems used for detecting or predicting seizures. The Supplementary Materials provided a concise overview of key attributes found in all the scrutinized studies (see Table S1). To synthesize the results of the review, the technical methodologies employed in HRV processing and clinical outcomes associated with different age-stratified populations were distinguished.

### 2.2. HRV Features

**Figure 2:**
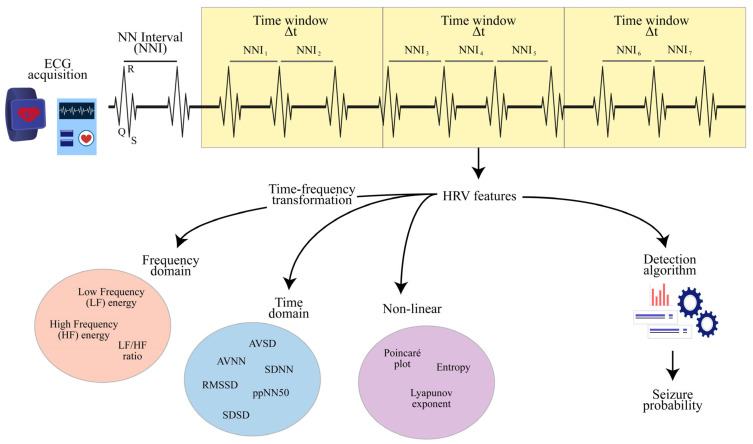
HRV extraction process. NNI = Normal-to-Normal Interval; LF = Low Frequency; HF = High Frequency; AVNN = Average of the NNI series; SDNN = Standard Deviation of the NNI series; AVSD = Average of the subsequent NNI difference series; SDSD = Standard Deviation of the subsequent NNI difference series; RMSSD = Root Mean Square of the subsequent NNI difference series; ppNN50 = percentage of NNIs differing more than 50 ms.

To estimate the HRV, it is first necessary to compute the normalized time intervals between consecutive R peaks, denoted as Normal-to-Normal Intervals (NNIs) [27]. The HRV can be derived from the entire NNI time series or, more commonly, a portion of the series. In the latter scenario, the HRV depends on a subset of the NNI samples and, consequently, becomes a time-dependent variable. For such a purpose, the common choice is to select a specific time window ∆t, so that any feature HRV(t, ∆t) is computed according to the NNIs measured within time t and t + ∆t. It has been shown that the selection of ∆t strongly affects the results of the HRV analysis [20], trading off between estimation accuracy and time sensitivity. A schematic representation of the HRV extraction process is given in Figure 2.

HRV metrics include three broad families, namely the time-based features, directly computed from the NNI series, the frequency-based features, computed from the spectrum of the NNI series, and the non-linear features [28]. The most common time-based features are the Average (AVNN) and the Standard Deviation (SDNN) of the NNI series. The latter denotes the tendency of NNI to keep a constant value and represents a naïve estimate for the HRV. From the NNI series, it is possible to extract the series SD of subsequent NNI differences ${SD}_{i}={NNI}_{i+1}-{NNI}_{i}$ and compute the Average (AVSD), the Standard Deviation (SDSD), and the Root Mean Square (RMSSD) of SD. Another time-based feature is the percentage (ppNNτ) of NNIs differing more than τ milliseconds during a specific interval, where τ is usually set to 50 ms or 20 ms. The RMSSD and ppNN50 are both recognized as two biomarkers of the vagal tone. More advanced time-based features include the width and the integral of the NNI distribution, where the latter is known as the triangular index.

To compute HRV metrics in the frequency domain, it is first necessary to implement a time-frequency transformation, using methods such as the Fast Fourier Transform (FFT) and the discrete wavelet transform [29,30]. The latter enables higher flexibility in terms of time-frequency accuracy and does not require the segmentation of the NNI series into windows. After estimating the HRV spectrum, the naïve choice is to compute the energy of significant frequency bands. According to the literature, the HRV’s low-frequency (LF) band reflects the combined sympathetic and parasympathetic influences on cardiac control, while the high-frequency (HF) band reflects parasympathetic and respiratory activity. Under these assumptions, the energy ratio between the LF and HF bands can be considered a measure of the sympathetic–vagal balance [31].

In the context of non-linear measures, the most popular tool is the Poincaré plot, which represents subsequent NNI values in a two-dimensional coordinate system where each point is given by a couple $\left({NNI}_{i+1},\;{NNI}_{i}\right)$ [32]. From the Poincaré plot, it is possible to compute the Cardiac Sympathetic Index (CSI), the Cardiac Vagal Index (CVI), and the modified Cardiac Sympathetic Index (mCSI) [33]. As denoted by the name, the CSI and the CVI are indicators of sympathetic and parasympathetic tones, while the mCSI is an extension of the CSI proving to be more sensitive to HRV changes. The NNI series can be characterized also via information-based metrics, such as entropy, which, in its original formulation, denotes the amount of information contained in an event [34]. Other approaches include the maximum Lyapunov exponent, a control theory’s measure denoting the predictability of a dynamic system [35], the recurrence quantification analysis, the detrended fluctuation analysis, and the correlation dimension, which all aim at characterizing the state space occupied by a time series [36].

### 2.3. Detection Algorithms

To detect seizure events, the naïve approach involves the definition of an alarm threshold over a specific HRV measure, considering a univariate analysis. A higher accuracy is achieved by using multivariate approaches, where different HRV features, also of different domains, are aggregated together. For this purpose, possible solutions include the naïve Bayes classifier, the linear or quadratic discriminant analysis, and the generalized linear models [37]. More advanced approaches include the implementation of supervised and unsupervised Machine Learning (ML) frameworks, where the first is designed to operate with labeled datasets and the latter enables the detection of new data patterns without human intervention [38,39]. Among the supervised ML tools, a popular solution is the Supporting Vector Machines (SVMs), which makes it possible to classify multivariate data into two or multiple classes by defining a hyperplane in the input space [40].

Independently of the technique implemented, the performance of any seizure detection framework can be described in terms of accuracy, sensitivity, and specificity. The accuracy is the probability for a detection system to correctly associate the algorithm input with its true class (seizure vs. non-seizure). Instead, the sensitivity and the specificity are defined as the probability of correctly detecting positive (seizure) and negative (non-seizure) events, respectively. Other performance metrics include the False Alarm Ratio (FAR), which is given by the number of false events reported within a specific period and, in the case of predictive systems, the anticipation time, which is the time shift between the alarm time and the seizure onset.

## 3. Results

The first research returned N = 402 different records, which were reduced to N = 86 after the double-screening procedure. Most of the records excluded in this phase focused on autonomic changes in animals, the probability of SUDEP, and the effects of vagus nerve stimulation. The text assessment led to the exclusion of N = 10 studies consisting of reviews of the literature and N = 4 studies that still did not comply with the review criteria. The final analysis included 72 studies that analyzed HRV during the peri-ictal phase, presented new seizure detection methods taking HRV measures as an input, or designed new wearable systems for such a purpose. A summary of the overall results is reported in Table 2.

### 3.1. HRV Features

To estimate HRV from Normal-to-Normal Intervals (NNIs), most of the analyzed works (N = 17) considered a time window ∆t lasting 5 min, which is recognized as a good trade-off between accuracy and time sensitivity [20]. In other cases, the value of ∆t was set to 4 min (N = 6), 3 min (N = 7), 2 min (N = 3), or 1 min (N = 5), better capturing rapid HRV changes at the cost of a reduced sample size. Finally, N = 9 studies considered a time window ∆t lasting even less than 60 s, maximizing the time sensitivity.

The Average (AVNN) and the Standard Deviation (SDNN) of the NNI series represented the most common HRV features and were exploited in N = 41 and N = 33 works among those analyzed, respectively. The authors of [71] suggested normalizing SDNN by instantaneous Heart Rate (HR) to reduce the bias of each patient on the analysis outcomes. The Root Mean Square Differences (RMSSDs) of the Subsequent NNI Difference (SD) series and the ppNN50, both recognized as indicators of vagal tone, were considered in N = 31 and N = 20 studies, respectively. Finally, a minority number of works estimated the HRV by the triangular index (N = 8), the width of the NNI distribution (N = 5), the Average of the SD series (AVSD) (N = 4), and the Standard Deviation of the SD series (SDSD) (N = 10). Interestingly, Behbahani and colleagues suggested considering a different lag index j for analyzing the SD series, so that the *i*-th element of the SD series is given by ${SD}_{i}\left(j\right)={NNI}_{i+j}-{NNI}_{i}$. This latter approach allows us to investigate the HRV changes in different time scales [72].

To analyze the HRV in the frequency domain, N = 19 of the analyzed works implemented Fast Fourier Transform (FFT). Other time-frequency transformations used in the review were the discrete wavelet transform (N = 6), the Wigner–Ville distribution (N = 5), the autoregressive models (N = 5), and the Welch algorithm (N = 5). Given the HRV spectrum, N = 46 studies quantified the energy of the LF and HF bands (and their ratio), while N = 6 studies detected specific HRV components without making assumptions about the band boundaries. Interestingly, a specific work was proposed to evaluate the HRV components according to their phase distribution by estimating a phase-locked spectrum [53]. Moreover, two works investigated the possibility that the EEG and ECG signals guide each other, estimating how the signal synchronization changes in time [54,55].

Focusing on the non-linear HRV metrics, a total of N = 22 works considered the Poincaré plot. A single work extended the Poincaré analysis by comparing the points $\left({NNI}_{i+j},\;{NNI}_{i}\right)$ for multiple lag indexes j [72]. A significant number of works (N = 10) estimated the complexity of the NNI series in terms of entropy. Among those works, N = 4 analyzed entropy in multiple time scales and two studies considered more specific entropy formulation, including the generalized entropy, the fuzzy entropy, the permutation entropy, the self-information, and the conditional entropy [43,51]. Finally, a limited number of studies (N = 5) characterized the NNI series via the recurrence quantification and the detrended fluctuation analysis, N = 4 studies considered the correlation dimension, and N = 3 studies implemented the maximum Lyapunov exponent.

### 3.2. Detection Algorithms

Among the works analyzed, N = 9 studies considered univariate approaches for seizure detection, while a single study implemented a heuristic classifier based on the Poincaré plot [56]. A limited number of works considered naïve Bayes classifiers (N = 3), linear or quadratic discriminant analysis (N = 6), and generalized linear models (N = 4) for discerning ictal and inter-ictal periods. Considering the ML approaches, most of the studies considered supervised models, including artificial neural networks (N = 2), the k-nearest neighbors algorithm (N = 4), random forests (N = 3), and Support Vector Machines (SVMs). This latter approach proved to be the most popular HRV-based solution for detecting seizures and was used in N = 10 studies among those analyzed. Finally, N = 3 works implemented unsupervised models, including different types of clustering techniques and the local outlier factor algorithm. An approach not falling in the previous definitions is given in [73,74], where ictal events were monitored through a multivariate statistical process control chart [112].

### 3.3. Neonatal Population

Among the reviewed studies, N = 8 works investigated HRV modifications during neonatal seizures. A first effort to quantify ictal autonomic changes in newborns is reported in [113], where the authors assessed a significant HR increase during neonatal seizures, without finding any significant HRV alterations. Two subsequent studies considered the energy in the HF and LF bands to discriminate ictal and non-ictal intervals [44,45], while another work proposed a seizure detection framework reaching an accuracy of about 85% using HRV metrics in the frequency domain [46]. More advanced seizure detection frameworks, considering information theory metrics for estimating the HRV, are reported in [42,43,47]. Frassineti and colleagues reached a detection accuracy of about 87% when using an SVM classifier taking multiscale entropy features as an input [41]. To further improve the detection performance, a single work combined EEG and HRV features via a data fusion approach, reaching a sensitivity and specificity of about 95% and 89%, respectively [48].

### 3.4. Pediatric Population

A total of N = 18 studies investigating autonomic abnormalities during the seizures of pediatric patients were reviewed. Two initial works, published in 2004 and 2005, analyzed HR in children with different epilepsy types, assessing that ictal tachycardia is more commonly associated with convulsive seizures [57,58]. Several studies (N = 6) denoted a correlation between seizures and sympathetic dominance, considering HRV metrics computed in both the time and the frequency domain [49,51,53,59,60,61]. A single study suggested that electrical seizures do not involve any HRV modifications [62], while Okanari and colleagues showed that children with post-ictal EEG suppression are associated with stronger sympathetic dominance during the pre-ictal phase [63]. More specific results are reported in [54,55], the authors of which jointly analyzed EEG and HRV signals, observing a stronger synchronization between the LF band of HRV and the Delta band of EEG during the peri-ictal periods. Moreover, two studies analyzed autonomic changes in a population including both children and adults [64,65], considering HRV metrics computed in the frequency domain. The results highlighted that the pediatric population is associated with stronger vagal suppression after tonic-clonic seizures than adult patients.

Two independent studies devised seizure detection systems, examined within a cohort including only children. Specifically, De Cooman and colleagues developed a detection algorithm for nocturnal seizures featuring autonomous adaptation to individual patient characteristics [56]. This system, tested on a group of 28 children, gained a sensitivity of 77.6% while keeping a False Alarm Ratio (FAR) of 2.56 events per night. Conversely, the authors of [52] compared different ML algorithms for detecting the pre-ictal transition in a sample of nine children. The best results were obtained with an SVM classifier with a multivariate input, yielding an accuracy of 77.1%.

### 3.5. Adult Population

In the adult population, N = 7 studies analyzed HRV changes during tonic-clonic seizures with Generalized (GTCS) or Focal to Bilateral (FBTCS) onset [75,76,77,78,79,80,114]. These studies assessed sympathetic dominance during the post-ictal periods independently of the seizure origin (temporal vs. extra-temporal) and highlighted how autonomic changes last longer in the case of convulsive than non-convulsive ictal events. In this regard, N = 3 other studies analyzed HRV during subclinical seizures, denoting that localized discharges are associated with minimal autonomic variation [81]. However, a stronger reduction in the parasympathetic tone is observed in case of the epileptic discharge spread within the network [66,82].

More heterogeneous results are obtained when analyzing the relationship between autonomic changes and seizure localization or lateralization, as performed in N = 12 studies in this review. An initial work documented a higher HR during seizures originating from the left temporal lobe [81], while another two studies suggested that ictal tachycardia does not correlate with the seizure onset [54,83]. Most of the studies in the literature suggested that stronger changes in autonomic cardiac control are associated with seizures of temporal origins, considering HRV measures computed in both the time and the frequency domains [84,85]. Two studies assessed an increase in the sympathetic tone during the pre-ictal periods in patients with both TLE and Frontal Lobe Epilepsy (FLE) [86,87], even in relation to motor events occurring during sleep.

In the case of seizure lateralization, the results are often contradictory. Two studies documented a reduction in the parasympathetic tone during the temporal seizures affecting the left lobe [88,89], while a third study assessed higher HRV and HR during the post-ictal phase of right-sided seizures [90]. Page and colleagues reported significant autonomic cardiac changes in terms of both HR and HRV, during seizures affecting both the temporal lobes [71]. Moreover, findings from N = 4 studies indicate that there is no significant variance in HRV parameters across ictal events with different lateralization [65,83,85,91]. Notably, it has been suggested that autonomic changes may serve as a biomarker for distinguishing between epileptic and psychogenic seizures [92,115]. Finally, Hödl and colleagues considered a population with Vagus Nerve Stimulation (VNS), observing that non-responding patients exhibit a stronger HRV reduction during the pre-ictal phase compared to the patients benefiting from the therapy [93,94].

Among the analyzed papers, N = 7 studies proposed tools for detecting focal seizures in the adult population. Jeppesen and colleagues designed a detection framework to discern ictal and non-ictal periods by monitoring reduction in parasympathetic activity [76,83,95,96]. By using the modified Cardiac Sympathetic Index (mCSI) to estimate the HRV, the designed system obtained a sensitivity of 100% in patients presenting significant tachycardia during the ictal phase. Alternative detection frameworks were proposed in [67,72,97], and the maximum performance (sensitivity of 100%) was obtained by Qaraqe and colleagues, who combined EEG and HRV features in a unique system.

Other studies (N = 7) analyzed differences among inter-ictal and pre-ictal intervals, suggesting that HRV changes are visible minutes before the seizure and, thus, enable an early prediction of ictal events. A case report from 2004 observed an increase in the HRV’s LF energy 12 min before seizures [98], while three studies found significant autonomic modifications during the 5 min preceding the ictal onset [99,100,101]. The authors of [68] implemented an SVM-based algorithm to identify the intervals (of 2 min duration) immediately preceding the seizure, obtaining a detection accuracy of 73%. Leal and colleagues considered a longer time horizon for characterizing the ictal transition and observed pre-ictal modifications up to 40 min before the seizure [102,103].

Taking advantage of the above evidence, N = 5 studies explicitly designed algorithms to predict seizure events from autonomic modifications. In this context, performance is assessed in terms of accuracy and anticipation time. Two studies designed prediction algorithms that ensured an anticipation time of 5 min [69,104], obtaining a maximum sensitivity of about 94% when using an SVM classifier taking multiple HRV features from different domains in input. Improved results are given in [50,74], where it is shown that a seizure can be predicted up to 15 min in advance without degrading the sensitivity levels. A recent study confirmed the large variability in the anticipation time, which could range from 3 to 30 min according to the target patient [70].

### 3.6. Wearable Systems

A total of N = 9 papers were reviewed analyzing seizure detection systems built with wearable devices, facing challenges related to the online estimation of HRV. Three studies analyzed systems to extract heartbeats in people with epilepsy automatically, without providing results in terms of detection accuracy [106,107,108]. In three consecutive studies, Jeppesen designed and refined a wearable system for detecting seizures during the activities of daily living [91,105,109]. The first version of the system led to a sensitivity of 87% with a FAR of 0.04/hour in patients, which was associated with ictal tachycardia. The adoption of a patient-adaptive algorithm further lowered the FAR by 31%, albeit with a slight decrease in sensitivity (78.2%). Running the algorithm on the data collected via an ECG patch, the authors obtained a sensitivity of about 92% and a FAR of about 0.1 events per hour in patients with marked ictal autonomic changes [110]. Finally, a recent work designed a system that integrates ECG, PPG, and EEG measures [111], leading to an accuracy of beyond 91% when detecting ictal events and status epilepticus. However, all the above solutions were tested offline and need further validation in phase III studies.

## 4. Discussion

This scoping review provides a comprehensive overview of the state-of-the-art role of cardiac autonomic variations, expressed through HRV, in the detection and prediction of ictal events. It is well known that numerous physiological phenomena related to immune, endocrine, metabolic, neurological, and cardiovascular functions present daily and multi-daily cycles. In individuals with epilepsy, seizure timing can be phase-locked to multi-day cycles in temperature, electrodermal activity, and heart rate [116]. A deeper understanding of the connections between seizures and the cardiovascular system could pave the way for innovative approaches to mitigate seizure risk, adapting clinical interventions, such as taking anti-seizure medication, to behavioral and sleep–wake patterns.

Undoubtedly, the detection of seizures is crucial for individuals with epilepsy (PwE), with varying implications depending on their age (Table 3). In newborns, the early detection of seizures is essential for timely clinical intervention, as untreated events can have significant and enduring impacts on the infant’s neurological development [19]. In older populations, the implementation of real-time warning systems can strongly enhance the QoL of both patients and their caregivers. From a practical perspective, seizure alarm systems would enable the caregiver to position the patient safely, protect them against injuries, and seek help. Such systems are particularly effective for nocturnal seizure monitoring, when ictal events are frequently unreported, and constitute a preventive measure against SUDEP [78].

Several devices using accelerometers (ACMs), surface electromyography (EMG), or multimodal recordings have been clinically validated for the detection of tonic-clonic seizures [21]. The detection of non-convulsive seizures remains challenging since it may entail only low-profile clinical signs. It has been shown that cardiac autonomic changes, assessed through HRV, could potentially serve as a useful biomarker for the detection of ictal events associated with minimal muscle contractions, undetectable via ACM or similar technologies [117]. Some studies have highlighted that cardiac autonomic changes anticipate the motor onset, thereby enabling the prediction of convulsive seizures, which demand increased assistance and may lead to critical consequences such as sudden death.

Studies analyzing autonomic changes during neonatal seizures highlighted that newborns exhibit lower inter-ictal HRV values than both children and adults. While full-term neonates exhibit an increase in vagal indexes of HRV during ictal events, the HRV does not show significant variations in preterm newborns [45], an effect that could be attributed to autonomic immaturity. To address such a limit, the computation of HRV features, both in time and frequency domains, should be tailored to the gestational and postnatal age of the newborns [19]. Because of the faster heart cycles in neonates, HRV dynamics can be characterized by using shorter time windows than those used for older populations, allowing a higher time sensitivity in assessing autonomic status. Particularly, a 2 min period has been suggested as the optimal window length for analyzing the NNI series in newborns, while, in the adult population, such a parameter is set to 5 min.

Several neonatal seizure detection methods have been designed and evaluated in real scenarios, leading to promising results for everyday use in clinical settings [42]. The affordability, non-invasiveness, and ease of use of ECG sensors make it possible to integrate such approaches as pre-screening tools in Neonatal Intensive Care Units (NICUs) to rapidly identify newborns at seizure risk, who may require further neurological investigation through continuous or amplitude EEG. In the context of newborn seizures, the maximum detection accuracy (87%) was obtained when using an SVM classifier taking multiple HRV features, including multi-scale entropy, as an input [41]. This result suggested that cardiac autonomic changes can be perceived only using advanced methodologies, including non-linear features for describing HRV evolution and ML algorithms for aggregating and processing the HRV measures.

In the context of the pediatric population, most studies observed that the peri-ictal phase is characterized by sympathetic dominance, a phenomenon that can be underlined using HRV measures in the frequency domain. This autonomic imbalance is more marked in children than adults, suggesting that seizure detection could be more efficient in younger populations. The heightened autonomic manifestations during seizures in children may stem from a lower threshold for epileptogenic activation of the central autonomic system, reflecting immature, less-established, subcortical seizure networks [65]. As occurred for the neonatal population, the best detection results (sensitivity of 77%) were obtained when implementing a multivariate SVM classifier taking multiple HRV features as inputs for discerning between ictal and inter-ictal periods.

Considering the adult population, it was shown that subclinical seizures lead to minimal autonomic variations [81], while convulsive seizures can be easily detectable from ECG signals. Interestingly, none of the studies in this review reported differences between geriatric and adult populations, suggesting that the same results also hold for older classes of people. In this regard, detecting convulsive seizures via HRV metrics could be non-relevant since other tools, including ACMs, may be used. The main utility of monitoring HRV lies in the possibility of predicting the motor onset, allowing the patient to gain a safe environment and caregivers to take proper measures to mitigate the seizure impact [118]. Besides leading to a strong enhancement in QoF, anticipating the ictal onset would make it possible to personalize medical treatments to specific seizure patterns and triggers, reducing the need for hospitalization and emergency care. The interval preceding seizure onset within which prediction becomes feasible exhibits an extraordinary variability, spanning from 30 min anticipation [70] to durations of about 5 min [69]. This considerable range in predictive timeframes highlights the intricacies involved in anticipating seizure events and underscores the need for meticulous investigation into the underlying physiological mechanisms.

Focal seizures have garnered increased attention in the field of HRV analysis, given the well-established connection between the autonomic system and TLE. HRV-based detection could prove to be a valuable tool for identifying and possibly predicting focal seizures that involve, at least partially, the structures of the temporal lobe. Autonomic modifications are not strictly related to TLE, since they may also occur in FLE and mostly rely on the involvement of the central autonomic network at seizure onset or propagation [119]. In general, the class of patients who enable efficient seizure detection and prediction is not well established [87]. Also, in this case, most works suggested modeling HRV according to frequency-based or non-linear metrics since time-based HRV features are often incapable of capturing the autonomic change in the peri-ictal phase [43].

In the last few years, increasing efforts have been directed at developing seizure detection algorithms that can be built with wearable devices. In this context, one of the most important performance parameters is the FAR. According to the ILAE recommendations, high FAR constitutes one of the major concerns of establishing a reliable wearable seizure detection device for non-convulsive seizures. While overlay-sensitive systems could erode patient confidence and lead to withdrawal from social activities, too many false alarms could discourage the patient adoption of these methods. To reduce FAR, it is important to consider patient-specific solutions, which can adapt detection thresholds and parameters to the HRV patterns of each patient, according to ML approaches [105]. Another critical point for minimizing false detections is to discern between different types of HRV changes, which might be related to trivial daily activities. In this context, it has been shown that advanced HRV metrics, e.g., based on the Poincaré Plot, can distinguish between exercise-induced ECG changes and the fast autonomic changes occurring during seizures [83].

In general, HRV-based algorithms, using ECG recorded with a wearable device, achieved good accuracy for detecting seizures only in patients with prominent ictal autonomic changes [91]. In the future, the individual pre-screening of HRV changes during a seizure remains necessary for determining if any form of HRV-based seizure detection system is feasible for the patient. Another limitation of the current literature is that it predominantly includes phase 2 studies running algorithms offline [109]. The validation of seizure detection tools in a real-life environment is still missing and should be the focus of the scientific community in the next few years. Finally, evidence has emerged that the combination of EEG and HRV features largely improves the seizure detection performance in terms of accuracy, false alarm rate, and latency compared with other methods [111]. A non-invasive wearable system that integrates ECG and EEG could represent the most promising tool for feasible seizure detection in the future. Following this path, the scientific and industry communities should address the challenges of designing new tools to collect multiple physiological signals, ensuring usability and acceptability in broad populations.

The main limitation of this scoping review is that it focuses on a single database (PubMed), and research works in scientific areas other than medical ones could have been missed. In addition, the research design led to the exclusion of papers not explicitly analyzing HRV modifications during the peri-ictal phase. A more comprehensive work, with less stringent review criteria, may identify other useful information, e.g., regarding the relationship between autonomic changes and antiepileptic drugs, for designing new methods for detecting and predicting seizure events.

## 5. Conclusions

The creation of tools aimed at anticipating ictal events represents a promising avenue for enhancing seizure management and the quality of life of PwE. This scoping review focuses on HRV analysis during the peri-ictal phase, emphasizing the potential of vegetative biomarkers for seizure control. Baseline HRV features in the time domain often miss vegetative variations related to ictal events. Optimal detection results come from leveraging advanced HRV features, such as energy in specific frequency bands or measures from the Poincaré Plot, using ML algorithms capable of aggregating diverse data. Detection performance is higher in neonatal and pediatric patients due to more significant cardiac autonomic changes, especially during seizures. However, most studies use datasets from clinical and controlled settings, with a limited exploration of HRV features from wearable devices. Out-of-hospital seizure detection appears feasible only for patients with substantial autonomic changes during the ictal transition. Currently, there is no evidence on clinical markers for accurately identifying patients and enabling reliable seizure detection. Establishing new guidelines to preemptively identify responsive patients is crucial for the widespread adoption of HRV-based detectors.

## Figures and Tables

**Table 1:** Search strategy. If a word ended with an asterisk character, all the search terms starting with that word were considered.

Index	Search	Results
1	“Epilepsy” [Mesh] OR “Seizures” [Mesh] OR “Epilep*” [title/abstract] OR “Seizure*” [title/abstract] OR “Ictal” [title/abstract] OR “Pre-ictal” [title/abstract] OR “Post-ictal” [title/abstract] OR “Peri-ictal” [title/abstract] OR “Inter-ictal” [title/abstract]	265,315
2	(“Time analysis” [title/abstract] OR “Power analysis” [title/abstract] OR “Nonlinear” [title/abstract] OR “Non linear” [title/abstract] OR “Non-linear” [title/abstract]) AND (“Electrocardiography” [Mesh] OR “Electrocardiography” [title/abstract] OR “Electrocardiogram” [title/abstract] OR “EKG” [title/abstract] OR “ECG” [title/abstract])	1847
3	“Heart rate variability” [title/abstract] OR “HR variability” [title/abstract]	23,489
4	#2 or #3	24,610
5	#1 and #4	402

**Table 2:** Summary of the results. AUC = Area Under Curve; CS = Convulsive Seizure; EMU = Epilepsy Monitoring Unit; ES = Electrographic Seizures; F(A+) = Focal seizures with autonomic changes; F(A−) = Focal seizures without autonomic changes; FAR = False Alarm Rate; IAS = Infantile Apneic Seizure; NA = Not Available; NCS = Non-Convulsive Seizure; NICU = Neonatal Intensive Care Unit; Sens = Sensitivity; Spec = Specificity; ↑ = best result; ↓ = worst result; + = 0–50% of the studies report the results; +++ = 75–100% of the studies report the results.

Population	Study Number	Population Size	Clinical Setting	Seizure Detection Performance	Seizure Prediction Time	HRV Changes			Seizure Type	References
						Pre-Ictal	Ictal	Post-Ictal		
Neonates (0–1 month)	8	Total = 256 Min = 5 Max = 52	NICU	↑ AUC = 87% [41] ↓ AUC = 62% [42]	NA	+	+++	+	Not specified	[41,42,43,44,45,46,47,48]
Infants (2–12 months)	1	7	EMU	NA	NA	+++	NA	+++	IAS	[49]
Children (1–18 years)	18	Total = 397 Min = 9 Max = 72	EMU	↑ Sens = 89.06% and FAR = 0.41/hour [50] ↓ Sens = 60.9% and Spec = 82.6% [51]	Min = 21.8 s [52] Max = 25 min [50]	+++	+++	+++	CS > NCS > ES	[49,50,51,52,53,54,55,56,57,58,59,60,61,62,63,64,65,66]
Adults (>18 years)	50	Total = 941 Min = 1 Max = 70	EMU	↑ Sens = 100.0% and FAR = 0.90/hour [67] ↓ Sens = 60.0% and Spec = 84.62% [68]	Min = 5 min [69] Max = 30 min [70]	+++	+++	+++ (CS > NCS)	CS > F(A+) > F(A−) > ES	[50,64,65,66,67,68,69,71,72,73,74,75,76,77,78,79,80,81,82,83,84,85,86,87,88,89,90,91,92,93,94,95,96,97,98,99,100,101,102,103,104]
			Wearable	↑ Sens = 93.10% and FAR = 0.04/hour [91] ↓ Sens = 78.20% and FAR = 0.03/hour [105]	NA	+++	+++	+++	CS > F(A+) > F(A−)	[73,91,105,106,107,108,109,110,111]
			ECG-Patch	Sens = 92.6% and FAR = 0.11/hour [110]	NA	+++	+++	+++	F(A+)	[110]

**Table 3:** Handy tips.

Handy Tips	
Neonates (0–1 month)	⮚ Ictal sympathetic activity is greater in full-term than pre-term newborns
Children (1–18 years)	⮚ Ictal sympathetic changes are greater in Convulsive (CS) than in Non-Convulsive Seizure (NCS) ⮚ Ictal HR and HRV modifications are greater in children than in adults
Adults (>18 years)	⮚ Peri-ictal HRV modifications are greater in CS as compared to NCS, particularly in the post-ictal phase ⮚ Ictal HRV changes might help differentiate epileptic seizures from non-epileptic episodes in individuals with PNES (Psychogenic Non-Epileptic Seizure) ⮚ Electrographic seizures are associated with minimal autonomic variations

## Supplementary Materials

The following supporting information can be downloaded at https://www.mdpi.com/article/10.3390/jcm13030747/s1, Table S1: List of the reviewed articles.
