# Supplementary material for: Heart Rate Variability as a Tool for Seizure Prediction: A Scoping Review
Source: J Clin Med. 2024 Jan 27;13(3):747. doi: 10.3390/jcm13030747 (PMC10856437; doi:10.3390/jcm13030747)
Supplement: Supplementary file 1 [file jcm-13-00747-s001.zip › jcm-2785727-supplementary.pdf]

Supplementary material

Table S1. List of the reviewed articles.

Article	Population	Seizure type	HRV features	HRV window	Clinical findings	Detection algorithm	Detection accuracy
[46]	52 newborns	Neonatal	Nonlinear	4 minutes			
[53]	5 newborns	Neonatal	Time-based, frequency-based	Unspecified	During the ictal phase, the sympathetic activity significantly increases		
[54]	28 newborns	Neonatal	Time-based, frequency-based	3 minutes	During the ictal phase, Heart Rate Variability (HRV) changes more significantly in full-term than pre-term newborns		
[55]	8 newborns	Neonatal	Time-based, frequency-based	Unspecified		Quadratic Discriminant Analysis (QDA), K Nearest Neighbors (K-NN), Naïve Bayes (NB)	Sensitivity = 85.70%<br>Specificity = 84.6%
[56]	51 newborns	Neonatal	Time-based, frequency-based, nonlinear	1-3 minutes		Support Vector Machine (SVM)	Area Under the Curve (AUC) = 62%
[57]	52 newborns	Neonatal	Time-based, frequency-based, nonlinear	4 minutes		Generalized Linear Model (GLM)	AUC = 69%
[58]	52 newborns	Neonatal	Time-based, frequency-based, nonlinear	4 minutes		Linear Discriminant Analysis (LDA), K-NN, Random Forest (RF), SVM	AUC = 87%
[59]	8 newborns	Neonatal	Time-based, frequency-based	Less than 1 minute		Neural Network (NN)	Sensitivity = 85.70%<br>Specificity = 84.6%<br>(HRV)<br>Sensitivity = 95.20%<br>Specificity = 94.3%<br>(HRV and EEG)
[65]	7 infants	Apneic	Time-based, frequency-based	5 minutes	During the ictal phase, the sympathetic activity significantly increases		
[43]	18 children	Various	Time-based, frequency-based, nonlinear	Unspecified	HRV significantly changes up to 100 seconds before the seizure onset		
[44]	18 children	Various	Frequency-based	Unspecified	During the ictal phase, the synchronization between HRV and EEG is stronger in right-sided Temporal Lobe Epilepsy (TLE)		

[45]	18 children	Various	Frequency-based	Unspecified	During the ictal phase, HRV and EEG signals are significantly synchronized		
[47]	37 children	Various	Time-based, frequency-based, nonlinear	5 minutes	During the pre-ictal phase, the sympathetic activity is stronger in generalized seizures		Sensitivity = 60.9% Specificity = 82.6%
[48]	28 children	Focal and Generalized Tonic-Clonic (GTC)	Time-based	Unspecified	During the ictal phase, Heart Rate (HR) is higher in GTC than in focal seizures	Heuristic classifier	Sensitivity = 77.60% False Alarm Ratio (FAR) = 2.56/night
[60]	20 children	Focal impaired awareness	Time-based	Unspecified	During the ictal and pre-ictal phase, HR significantly increases		
[61]	37 children	Various	Time-based	Unspecified	During the ictal phase, HR is higher in generalized seizures		
[62]	8 children	Absence seizures	Time-based	2 minutes	During the ictal phase, HR significantly increases		
[63]	40 children	Various	Time-based, frequency-based	Unspecified	During the ictal phase, the sympathetic activity is stronger in refractory than non-refractory epilepsy		
[64]	40 children	Various	Time-based, frequency-based	5 minutes	During the ictal phase, the sympathetic activity significantly increases		
[66]	73 children	Subclinical	Time-based	Less than 1 minute	During the ictal phase, the sympathetic activity is stronger in older children		
[67]	35 children	GTC	Time-based, frequency-based	3-5 minutes	During the pre-ictal phase, HRV changes more significantly in the case of postictal generalized EEG suppression		
[70]	9 children	Focal	Time-based, frequency-based	Less than 1 minute		NB, K-NN, LDA, RF, GLM, SVM	Accuracy = 77.1 %
[68]	13 adults, 7 children	GTC	Frequency-based	Unspecified	During the post-ictal phase, the sympathetic activity is stronger in children than in adults		
[69]	46 adults, 53 children	Focal	Time-based	Less than 1 minute	During the ictal phase, HR and HRV change more significantly in children than adults		

[79]	6 adults, 5 children	Subclinical	Time-based, frequency-based	Less than 1 minute	During the ictal phase, the sympathetic activity is stronger in generalized than focal seizures		
[107]	15 adults and children	Focal, Focal to Bilateral Tonic-Clonic (FBTC), GTC	Time-based, frequency-based	Unspecified	HRV significantly changes up to 25 minutes before the seizure onset	SVM	Sensitivity = 89.06% FAR = 0.41/hour
[41]	13 adults	Various	Time-based, frequency-based, nonlinear	4 minutes	During the ictal phase, HRV is lower in bitemporal seizure		
[42]	16 adults	Focal and FTBTC	Time-based, frequency-based, nonlinear	5 minutes		Heuristic classifier	Sensitivity = 80.42% FAR = 0.15/hour
[49]	7 adults	Focal	Time-based, frequency-based	3 minutes		Multivariate Statistical Process Control (MSPC)	Sensitivity = 85.70% FAR = 0.62/hour
[50]	14 adults	Focal	Time-based, frequency-based	3 minutes	HRV significantly changes up to 5 minutes before the seizure onset	MSPC	Sensitivity = 91% FAR = 0.7/hour
[71]	25 adults	Focal impaired awareness and FTBTC	Time-based	Less than 1 minute	During the post-ictal phase, HRV decreases more significantly in FTBTC than focal seizures		
[72]	11 adults	GTC	Time-based, frequency-based, nonlinear	5 minutes	During the pre-ictal phase, the parasympathetic activity significantly increases		
[73]	30 adults	GTC and FTBTC	Time-based	Unspecified	During the post-ictal phase, HRV significantly decreases		
[74]	40 adults	Focal and GTC	Time-based, frequency-based, nonlinear	More than 5 minutes	During the ictal phase, the sympathetic activity is stronger in GTC than in focal seizures		
[75]	31 adults	Various	Time-based, frequency-based	5 minutes	During the post-ictal phase, HRV decreases more significantly in GTC seizures		
[76]	70 adults	Various	Time-based, frequency-based	1 minute	During the ictal phase, HRV changes more significantly in convulsive than non-convulsive seizures		
[78]	26 adults	Subclinical	Time-based	Less than 1 minute	During the ictal phase, HR is higher in the left-sided TLE than in Frontal Lobe Epilepsy (FLE)		

[80]	4 adults	Generalized non-convulsive	Frequency-based	Unspecified	During the ictal phase, the sympathetic/parasympathetic balance significantly changes		
[81]	17 adults	Focal and FTBTC	Frequency-based, nonlinear	Less than 1 minute	During the pre-ictal phase, the sympathetic activity is stronger in FTBTC than in focal seizures		
[82]	42 adults	Focal	Time-based, frequency-based	5 minutes	During the ictal phase, HR and HRV change more significantly in people with TLE		
[83]	23 adults	TC	Time-based, frequency-based	1-5 minutes	During the post-ictal phase, the sympathetic activity significantly increases		
[84]	10 adults	Nocturnal frontal lobe	Time-based	Unspecified	During the pre-ictal phase, the sympathetic/parasympathetic balance significantly changes		
[85]	58 adults	Focal	Time-based, frequency-based	5 minutes	During the post-ictal phase, the sympathetic activity is stronger in TLE seizures		
[86]	14 adults	Various	Time-based, frequency-based, nonlinear	4 minutes	During the ictal phase, the parasympathetic activity is stronger in left-sided than right-sided TLE		
[87]	16 adults	Focal and FTBTC	Time-based, frequency-based, nonlinear	5 minutes	During the ictal phase, sympathetic activity is stronger in right-sided than left-sided seizures	SVM	Sensitivity = 83.13%<br>Specificity = 90.36%<br>(right-sided)<br>Sensitivity = 76.47%<br>Specificity = 82.35%<br>(left-sided)
[87]	14 adults	Focal and FTBTC	Time-based, frequency-based	5 minutes	During the post-ictal phase, HRV changes more significantly in FTBTC seizures		
[88]	25 adults	Focal	Time-based, frequency-based, nonlinear	Unspecified	During the post-ictal phase, the parasympathetic activity is stronger in right-sided than left-sided seizures		
[89]	43 adults	Focal and FTBTC	Nonlinear	Unspecified		Threshold-based	Sensitivity = 93.10%<br>FAR = 0.04/hour
[90]	50 adults	Focal impaired awareness and psychogenic	Time-based, frequency-based, nonlinear		During the ictal phase, the sympathetic activity is stronger in epileptic		

					than in psychogenic seizures		
[92]	22 adults	Vagus Nerve Stimulation (VNS)	Time-based, frequency-based, nonlinear	Unspecified	During the ictal phase, HRV is lower in VNS non-responders than in VNS responders		
[93]	7 adults	VNS	Time-based, frequency-based, nonlinear	Unspecified	During the pre-ictal phase, the parasympathetic activity significantly increases		
[94]	6 adults	Various	Frequency-based	Unspecified	During the ictal phase, the sympathetic activity significantly increases		
[95]	5 adults	Focal impaired awareness	Nonlinear	Unspecified	During the ictal phase, the sympathetic activity significantly increases		
[96]	16 adults	Focal and FTBTC	Time-based, frequency-based, nonlinear	4-5 minutes		Threshold-based	Sensitivity = 78.59%<br>FAR = 0.21/hour
[97]	10 adults	Various	Time-based, frequency-based	Unspecified		SVM	Sensitivity = 100.00%<br>FAR = 0.90/hour
[98]	1 adult	GTC	Frequency-based	3 minutes	During the pre-ictal phase, the sympathetic activity significantly increases		
[99]	12 adults	Focal and FTBTC	Time-based, frequency-based	Unspecified	During the ictal phase, HRV significantly decreases		
[100]	24 adults	Focal and FTBTC	Frequency-based, nonlinear	5 minutes	During the pre-ictal phase, the sympathetic activity is stronger in males than females		
[101]	9 adults	Focal	Time-based	Unspecified	HRV significantly changes between 6.5 and 3.5 minutes before the seizure onset		
[102]	12 adults	Various	Time-based, frequency-based, nonlinear	1-2 minutes	HRV significantly changes up to 2 minutes before the seizure onset	SVM	Sensitivity = 60.0%<br>Specificity = 84.62%
[103]	37 adults	Focal	Time-based, frequency-based, nonlinear	5 minutes	HRV significantly changes up to 50 minutes before the seizure	LDA	Accuracy = 88.04%
[104]	41 adults	Various	Time-based, frequency-based, nonlinear	5 minutes	HRV significantly changes up to 40 minutes before the seizure	Unsupervised clustering	

[105]	7 adults	Various	Time-based, frequency-based, nonlinear	5 minutes	HRV significantly changes up to 10 minutes before the seizure onset	Threshold-based	Sensitivity = 88.30% Specificity = 86.2%
[106]	12 adults	Various	Time-based, frequency-based, nonlinear	1-3 minutes	HRV significantly changes up to 5 minutes before the seizure onset	SVM	Sensitivity = 94.10% FAR = 0.49/hour
[108]	5 adults	Various	Time-based, frequency-based, nonlinear	2-3 minutes	HRV significantly changes from 3 to 30 minutes before the seizure onset	SVM, Local Outlier Factor (LOF)	Sensitivity = 93.0% Specificity = 95.8%
[109]	14 adults	Various					
[110]	56 adults	Various					
[111]	10 adults	No					
[112]	19 adults	Focal and GTC	Nonlinear	Unspecified		Threshold-based	Sensitivity = 87.00% FAR = 0.04/hour
[113]	62 adults	Various	Nonlinear	Unspecified		GLM	Sensitivity = 78.20% FAR = 0.03/hour
[114]	6 adults	Various	Nonlinear	Unspecified		Threshold-based	Sensitivity = 92.60% FAR = 0.11/hour
[115]	10 adults	Various	Time-based, frequency-based, nonlinear	Less than 1 minute		NB, LDA, SVM, K-NN, RF, NN	Sensitivity = 85.4% Specificity = 93.1%
